# Adaptation of a parasitic lifestyle by *Cuscuta* gronovii Willd. ex Roem. & Schult.: large scale gene deletion, conserved gene orders, and low intraspecific divergence

**DOI:** 10.1080/23802359.2021.1911702

**Published:** 2021-04-20

**Authors:** Yang Ni, Mei Jiang, Haimei Chen, Linfang Huang, Pinghua Chen, Chang Liu

**Affiliations:** a Key Laboratory of Ministry of Education for Genetics, Breeding and Multiple Utilization of Crops, National Engineering Research Center of Sugarcane, College of Agriculture, Fujian Agriculture and Forestry University, Fuzhou, P. R. China; b Key Laboratory of Bioactive Substances and Resource Utilization of Chinese Herbal Medicine from Ministry of Education, Engineering Research Center of Chinese Medicine Resources from Ministry of Education, Institute of Medicinal Plant Development, Chinese Academy of Medical Sciences, Peking Union Medical College, Beijing, P. R. China

**Keywords:** *Cuscuta gronovii*, SNP, RNA editing site, analysis, markers

## Abstract

*Cuscuta gronovii* Willd. ex Roem. & Schult. is a parasitic plant and widely distributed in Europe and North America. Here, we compared the plastome sequences of a *C. gronovii* plant collected from China and other 16 *Cuscuta* plastomes, including one *C. gronovii* plastome from North America. The plastome was 86,727 bp in size with a pair of inverted repeats (IRs) of 14,354 bp, a large single-copy (LSC) region of 50,956 bp, and a small single-copy (SSC) region of 7,063 bp. We predicted 97 genes in the plastome, including 61 protein-coding genes, eight ribosomal RNA genes, and 28 transfer RNA genes. We detected a total of 21 microsatellite, 16 tandem, and ten interspersed repeats in the genome. Gene contents analysis of 17 *Cuscuta* plastomes showed the loss of the entire *ndh* gene family. The phylogenomic analysis using 22 shared protein sequences shows that the two *C. gronovii* formed a cluster. Thirteen of *Cuscuta* plastomes showed no rearrangement. The other three showed smaller inversions and smaller numbers of gene deletion. Next, we identified the two INDELs, one SNP site between the two *C. gronovii* plastomes. And we identified two putative RNA editing sites. Lastly, to distinguish between *C. gronovii* and two medicinal *Cuscuta* species, *C. chinensis* and *C. australis*, we identified ten molecular markers based on the genome sequences. The overall large-scale gene loss, conserved gene order, low intraspecific divergence are consistent with the adaptation of C. *gronovii* to a parasitic lifestyle.

## Introduction

The *Cuscuta* spp. (dodders) belongs to the genus *Cuscuta* (*Convolvulaceae*, *Solanales*), which contains about 200 plant species, most of which are parasitic (Dawson et al. 1994). *Cuscuta* spp. exhibit massive body plan changes, being leaf- and rootless throughout their life cycles (Sun et al. 2018). The adaptation of the parasitic lifestyle appears to be a continuous process (Cooke and Black 1987). The gradual loss of the chloroplast contents and activities coincide with the degree of parasitism. Based on the chloroplast morphology and photosynthetic capacity, the *Cuscuta* spp. can be divided into three groups. The first group has functional chloroplasts and photosynthetic capacity. A second group has degenerated chloroplasts with restricted photosynthetic activity. A third group are devoid of chloroplasts and are achlorophyllous.

Photosynthesis is a key biological process in plants that are carried out by the organelle chloroplasts. As a result, the loss of chloroplast and photosynthetic activity is critical in adapting to the parasitic lifestyle. The chloroplast genome (plastome) is a circular DNA molecule and encodes proteins, which plays an essential role in photosynthesis (Keeling 2004). One manifestation of the loss of the photosynthetic activity is the deletion of genes from the plastomes. As a result, the plastomes' content and structure were often used to study the parasitic plants' evolution and adaptation.

*C. gronovii* Willd. ex Roem. & Schult. (the plantlist.org, last accessed: 17 August 2020) is initially distributed in Europe and North America. A previous study showed that the plastid genome of *C. gronovii* had suffered extensive deletions by comparing the relative sizes and coding capacities of the plastid DNAs of three *Cuscuta* species based on hybridization patterns with tobacco plastid DNA (Berg et al. 2003). Funk et al. compared the plastome differences between *C. gronovii* and *Cuscuta reflexa*, found (a) the parallel losses of genes for the subunits of the plastid-encoded RNA polymerase and its promoters; (b) the loss of *mat*K gene, a putative splicing factor; and (c) a significant reduction of RNA editing (Funk et al. 2007).

Our original goal is to study *C. australis’* parasitism by analyzing its plastome’s content and structure. *C. australis* is a species listed in the Chinese Pharmacopeia. However, sequence comparison with those from the database suggests that what we have sequenced is most likely to be *C. gronovii*. As a result, we carried out a systematic comparative genomic study of *C. gronovii* plastome and those from another sixteen *Cuscuta* species. We also characterized the intraspecific variations, including Insertion/deletions (INDELs), SNPs, and post-transcriptional modification events such as RNA-editing. Overall, the levels of genome deletion, rearrangement, intraspecific variation, and post-transcriptional modifications are consistent with the adaptation of the *Cuscuta* species to a parasitic lifestyle. The results provide valuable information regarding the gradual transformation of parasitism for *Cuscuta* species.

## Materials and methods

### Plant material, DNA extraction, and sequencing

The fresh stems of *C. gronovii* were collected from the Institute of Medicinal Plant Development (IMPLAD), Beijing, China. Professor Zhao Zhang of IMPLAD identified the samples. The genomic DNA was extracted using a Plant Genomic DNA kit (Tiangen Biotech, Beijing, China) and stored at the Herbarium of IMPLAD with accession number: Implad20170662. The DNA library was constructed with 1ug DNA using the library preparation kit (New England BioLabs, America) and was sequenced on a Hiseq 2500 platform (Illumina, San Diego, CA, USA).

### Genome assembly, validation, and annotation

The raw data pre-processing was conducted with two procedures by the Trimmomatic software (Bolger et al. 2014). First, we cut the adapters for the base reads. The sequence quality standard is then set at quality values of Q < 19 and with more than 5% bases being ‘N’ according to the reads. The plastome was de novo assembled using NOVOPlasty (v.4.0) (Dierckxsens et al. 2016). We employed 14 *Cuscuta* plastomes as reference sequences (Supplementary Table S1). We used Gepard software to draw Dot plots between the assembled genome and the reference genome to identify the plastome structure (Krumsiek et al. 2007). To validate the assembly’s correctness, we mapped sequence reads to the assembled genome and then examined and edited the mapping results using the consed software (Gordon et al. 1998). We used CpGAVAS2 to annotate the plastome. We manually edited the annotations with problems using the Apollo (Misra and Harris 2005) software. The genome sequence and annotation results have been submitted to the GenBank with accession number MT872375. In the following text, *C. gronovii* plastome refers to the MT872375 sequence without otherwise specified.

### Characteristics and repeat analysis

We used modules embedded in the CpGAVAS2 webserver to analyze codon usage and repeat contents. We identified the microsatellite sequences using Misa software (Sebastian et al. 2017). We identified the tandem repeats using TRF software (Benson 1999) with the size of the repeat unit ≥7. We calculated the GC content using Editseq from the DNASTAR Lasergene package (v9) (Burland 2000). We identified the interspersed repeats using VMATCH software (Kurtz et al. 2001).

### Phylogenetic analysis

The plastome sequences of 16 *Cuscuta* species and two outgroup species (*Ipomoea batatas* and *Ipomoea purpurea*) were downloaded from GenBank. In total, we found 22 shared DNA sequences (*acc*D*, atp*A*, atp*B*, atp*H*, clp*P*, rpl*2*, rpl*14*, rpl*16*, rpl*20*, rpl*22*, rpl*33*, rps*2*, rps*3*, rps*4*, rps*7*, rps*8*, rps*11*, rps*12*, rps*15*, rps*18*, rps*19*, ycf*2) in the 19 plastomes and aligned them with MAFFT software (Katoh et al. 2005). ‘TVM + F+G4’ was found to be the best evolutionary model, according to BIC (Bayesian Information Criterion) scores. The phylogenetic tree of *C. gronovii* and its closest relatives were built using the IQ-TREE with this model (Nguyen et al. 2015). We used UBBoot to perform the bootstrap analysis with 1000 replicates.

### *Gene deletion identification of* Cuscuta genus

We constructed a blastable database of all plastome sequences of the *Cuscuta* genus using the tool formatdb. The gene belongs to the *ndh* gene family from *Arabidopsis thaliana* were selected as the query to search the database using BLAST with the e-value cutoff of 1e-10 and alignment length of more than 50 bp (Madden 2013).

### *Discovery of intraspecific divergence between the* C. gronovii *plastomes*

Previously, another *C. gronovii* plastome has been published (NC_009765) (Funk et al. 2007). The two plastome sequences were aligned using the module seqman from the DNASTAR Lasergene package (v9) (Burland 2000) with default parameters. The INDELs and SNPs were then identified manually.

### *Identification of RNA editing sites in* C. gronovii *plastome*

We downloaded the raw data (SRR SRP200788) of RNA-seq experiments for *C. gronovii* from the GenBank SRA database (http://www.ncbi.nlm.nih.gov/sra). The dataset contains reads from the stems of three biological replicates. RNA editing sites were analyzed using REDItools (Picardi et al. 2015) with the following parameters: coverage ≥ 5, frequency ≥ 0.1, and *p*-value ≤ .05.

### Molecular markers development

To identify molecular markers to distinguish three species, we used ecoprimer to identify primers based on the reference genome sequences with the following parameters: -l 300 -L 600 -e 0 − 3 2 -t species -T 1 -U -f -O 25. Ecoprimers tend to predict many primer pairs, many of which overlap with each other. These overlapping primer sequences were merged to longer sequences for primer design using custom scripts.

## Results

### Validation of the assembly and general features of the plastome

We assembled the raw data using the chloroplast genome sequence in the Ref-seq database as reference (Supplementary Table S1). The assembly results of ten of them are in complete agreement. However, the assembly using the four chloroplast genome sequences of *Cuscuta reflexa* (NC_009766.1), *Cuscuta australis* (NC_045885.1), *Cuscuta obtusiflora* (NC_009949.1), *Cuscuta exaltata* (NC_009963.1 showed different result. The alignment of the assembly results obtained using the different reference sequences were shown in supplementary file 1. We eventually chose the majority consensus results for the analysis.

The entire length of the *C. gronovii* plastome is 86,727 bp, significantly shorter than most plastomes in GenBank. To confirm the correctness of the assembly, we first compared the *C. gronovii* plastome with those of *A. thaliana* (Supplementary Figure S1) and itself (Supplementary Figure S2). All the Dot plots have a diagonal line and two perpendicular lines, representing the inverted repeats (IRs). The plastome of *C. gronovii* is largely colinear with that of *A. thaliana*, although it is significantly shorter. Then we mapped the reads to the assembly. There are no low coverage sites observed through the plastome (Supplementary Figure S3). The junction site result shows that the IR region’s coverage depth is two times that of the LSC region (Supplementary Figure S4, S5). The above results support the high-quality of our plastome assembly.

**Figure 1.**
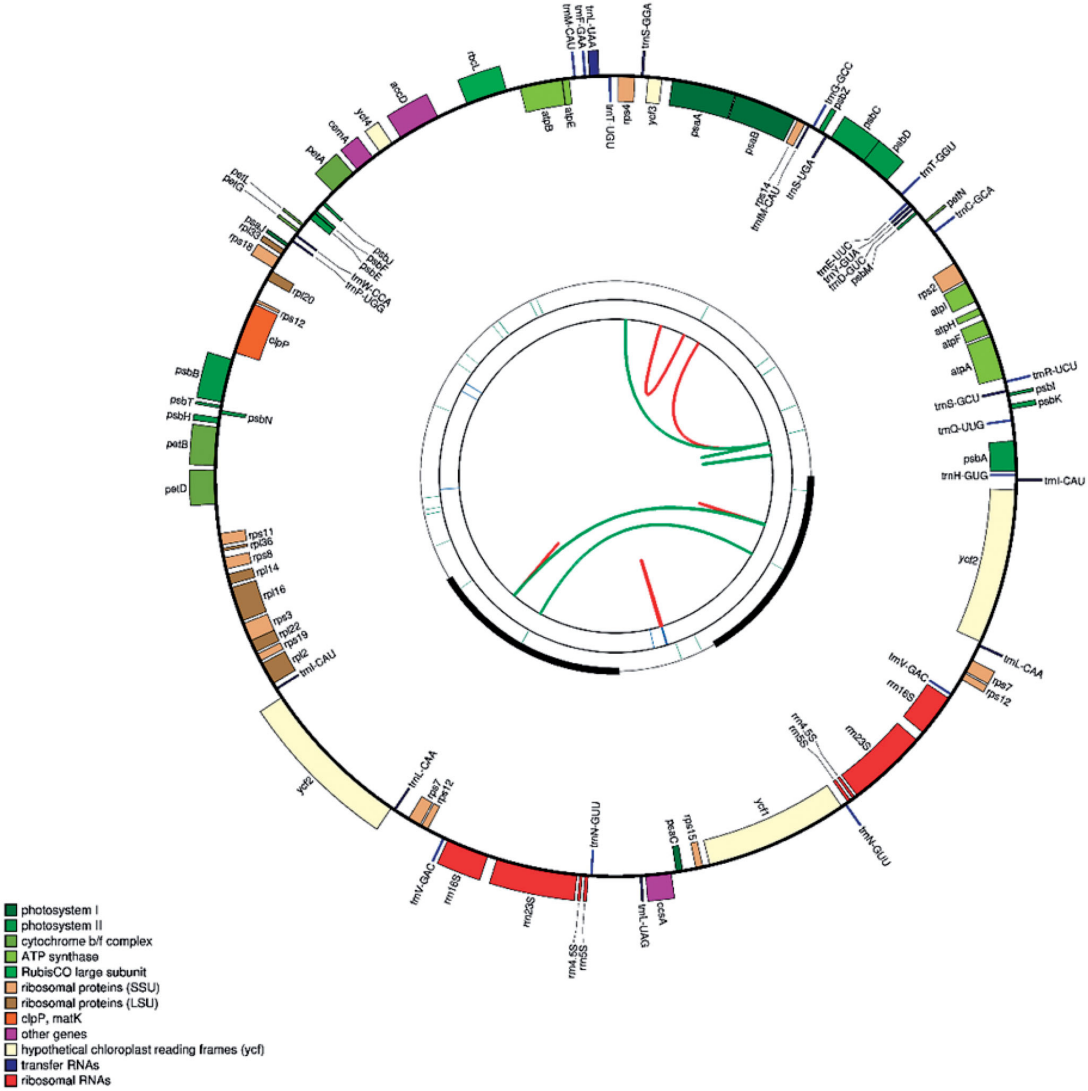
The schematic representation of the plastome of *C. gronovii* created using CPGAVAS2. The map contains four rings. From the center going outward, the first circle shows the forward and reverse repeats connected with red and green arcs, respectively. The next circle shows the tandem repeats marked with short bars. The third circle shows the microsatellite sequences identified using MISA. The fourth circle shows the gene structure on the plastome. The genes were colored based on their functional categories, which are shown in the left corner.

The plastome of *C. gronovii* has a typical quadripartite structure, consisting of a pair of IR regions of 14,354 bp, a large single-copy (LSC) region of 50,956 bp, and a small single-copy (SSC) region of 7,063 bp (Figure 1). The genome encodes 97 genes, including 61 protein-coding genes, eight ribosomal RNA (rRNA) genes, and 28 transfer RNA (tRNA) genes (Supplementary Table S2). Among these genes, three genes (*pet*B, *pet*D, *rpl*16) contain one intron, one gene *clp*P contains two introns, and one tRNA gene (*trn*L-UAA) contains one intron (Supplementary Table S3). The length of the protein-coding sequence (CDS) in the plastome is 49,104 bp, representing 56.62% of the plastome sequence’s total length. In contrast, the rRNA and tRNA genes’ size is 8,878 bp and 2,121 bp, representing 10.24% and 2.45% of the plastome sequence’s entire length. The GC content analysis showed that the overall GC content is 37.72%, whereas those for the tRNA genes, rRNA genes and for the protein-coding regions are 51.01%, 54.79%, and 37.66%, respectively.

Moreover, a total of 22,437 codons were identified in the plastome of *C. gronovii*. Among these codons, 3,509 codons encode leucine, and 336 codes encode cysteine, respectively, representing the most and least abundant coded amino acids in the *C. gronovii* plastome (Supplementary Table S4).

### Gene pseudonization and deletion

**Figure 2.**
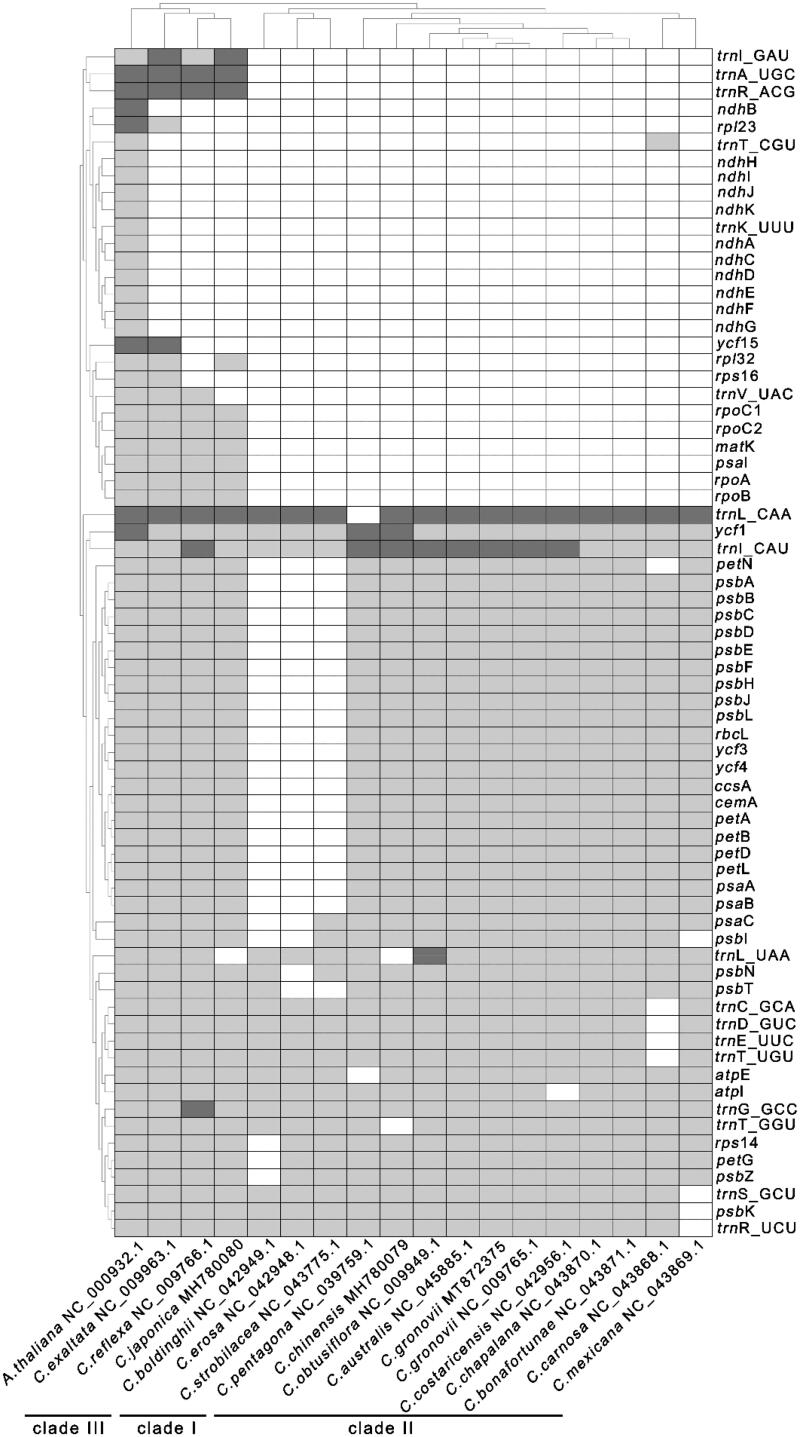
Gene deletion in *Cuscuta* genus. Each column represents a *Cuscuta* species. Each row represents a gene. The cells are colored asdark gray, light gray, and white, which represents two, one, and zero-copy of genes.

To explore the *Cuscuta* species’ gene deletion scale, we performed a two-way clustering analysis of 16 *Cuscuta* species and use *A. thaliana* as a reference (Figure 2). The tRNA (*trn*K-UUU) and genes from the *ndh* family (*ndh*A,*ndh*B, *ndh*C*, ndh*D*, ndh*E*, ndh*F*, ndh*G *ndh*I*, ndh*J*, ndh*K) were lost in all *Cuscuta* plastomes compared with that of *A. thaliana.* Based on the scale of gene deletion, the 16 *Cuscuta* species can be divided into three groups. *C. japonica*, *C. reflex*, and *C. exaltata* form one group. They consisted of three tRNA genes (*trn*I-GAC, *trn*A-UGC, *trn*R-ACG) and six protein-coding genes (*rpo*A, *rpo*B, *rpo*C1, *rpo*C2, *psa*l, *mat*K) that are missing from the other *Cuscuta* species. *C. boldinghii,C. erosa, C. strobilacea* form one group. There are 21 common protein-coding genes (*pet*A, *pet*B, *pet*L, *pet*N, *psa*A, *psa*B, *psb*A, *psb*B, *psb*C, *psb*D, *psb*E, *psb*F, *psb*G, *psb*H, *psb*J, *psb*K, *rbc*L, *ycf*3,*ycf*4,*ccs*A,*cem*A) are missing in those species.And the others form another group. There is no difference in the gene contents between *C. gronovii* (MT872375) and *C. gronovii* (NC_009765). The results show that there is lower intraspecific diversity in terms of gene contents. To further examine the gene loss in the *ndh* gene family of *Cuscuta* spp, we searched the *Cuscuta* spp sequences with those from *A. thaliana* using BLAST. We identified large fragments of the *ndh*B genes in the plastome sequences of *C. reflexa and C. exaltata* (Supplementary Figure S6 and S7). Compare to the *ndh*B gene in *A. thaliana*, we found a base G changed to T at position 22 resulted in the appearance of a stop codon in the *ndh*B coding sequence (CDS) in *C. reflexa* (Supplementary Figure S8). Similarly, a stop codon appeared in the CDS of *ndh*B at position 87–90 in *C. exaltata* due to a large indel of 25 bp (Supplementary Figure S9).

### Repeat analysis

Microsatellites, also known as simple sequence repeats (SSR), are abundant across genomes and show high polymorphism (Li et al. 2002). Microsatellite repeats, tandem repeats, and interspersed repeats were detected in the plastome. A total of 21 microsatellite repeats (A/T) was detected (Supplementary Table S5). All the microsatellite repeats are less than 100 bases. And eight microsatellite repeats were located in the CDS regions (*atp*F*, rps*7, *cem*A, *ycf*1, *ycf*2). Using the cutoff 90% similarity between repeat units, we detected 16 repeats in the plastome of *C. gronovii* finally (Supplementary Table S6). The lengths of repeat units range from 11 bp to 135 bp. For interspersed repeats, four palindromic repeats and six direct repeats were identified (Supplementary Table S7). The most extended interspersed repeat unit is 36 bp long and located in the intergenic region of *psb*I and *trn*S-GCU. In the future, when more plastome sequences become available, these repeats can be compared, and hopefully, some of them will serve as the polymorphic markers for intraspecific discrimination.

### Phylogenetic analysis

**Figure 3.**
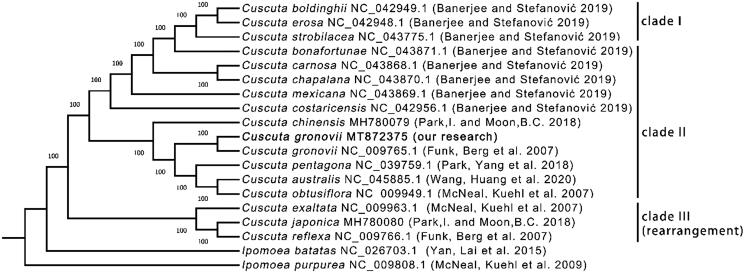
The phylogenomic tree of species from genus constructed using the maximum likelihood (ML) method. The number next to each node represents the bootstrap values. The GenBank accession numbers are shown after the Latin names. The sequence obtained from this study was highlighted in Bold and noted with ‘our research.’ The clade I, II and III are the same as those shown in Figure 2. The clade III containing three three species whose plastom sequences conain several rearranged regions.

We selected 16 *Cuscuta* species to construct the phylogenetic tree. *I. batatas* and *I. purpurea* were selected as the outgroup taxa. The complete plastome sequences (Supplementary Figure S10A), all CDS sequences (Supplementary Figure S10B), all s hared gene protein sequences (Supplementary Figure S10C) and all shared gene nucleic acid sequences (Figure 3) were used to construct four trees. All the trees have the identical topology. However, the tree constructed with all shared gene nucleic acid sequences had the highest bootstrap values. According to the phylogenetic analysis, these *Cuscuta* species formed two clades. *C. exaltata*, *C. japonica*, and *C. reflexa* belong to one clade, and the others belong to another clade, consistent with previous results from the gene deletion analysis. As expected, two *C. gronovii* were clustered together. Furthermore, the bootstrap scores at all nodes in the phylogenetic tree were 100, indicating the tree's high reliability.

### Snp identification from the plastome

To discover SNPs from the plastome, we compared the two plastomes of *C. gronovii* and identified 2 INDELs and 1 SNP (Supplementary Table S8). Among the INDELs, the first one is an 18 bp fragment inserted in the IGS region between the genes *trn*S-UGA and *psb*Z. The second one is located in the IGS region between the genes *rbc*L and *acc*D. The SNP located in the CDS of gene *acc*D is from C to A, causing the encoded amino acids to change from Asp to Glu. The molecular markers based on the intergenic region between the *trn*S-UGA and *the psb*Z gene might help distinguish *C. gronovii* under the species level.

### *Identification of RNA editing sites in* C. gronovii *plastome*

To determine the possible presence of RNA editing events in the *C. gronovii* plastid, we downloaded the RNA-seq data from the Public database and compared them with the plastome sequence. The RNA-seq data were obtained from the stems of *C. gronovii*. We identified two putative RNA editing sites using the REDItools software (Supplementary Table S9). The corresponding editing events cause one G to A transition and one U to C transition. The two RNA editing sites are located in the coding regions of gene *rrn*4.5S. Since *rrn*4.5S does not encode any protein, it is unclear if these RNA-editing sites will have any functional consequence. It appears that RNA-editing is not used to correct the miss-coding of proteins in the chloroplast of *C. gronovii*.

### *Structural variation of* Cuscuta *plastomes*

All the *Cuscuta* plastome sequences in GenBank (Last accessed: 31 December 2020) were compared with that of *A. thaliana* for structural variation analysis. All the Dotplot results showed a high degree of colinearity between the *Cuscuta* plastomes and *A. thaliana* (Supplementary Figure S1 and Figure S11A-P). It should be noted that the starting positions of these *Cuscuta* plastomes were set differently. The SSC region's first position was selected as the starting position for *C. bonafortunae, C. carnosa, C. boldinghii, C. mexicana, C. erosa, C. strobilacea,C. costaricensis* and *C. chapalana.* In contrast, the LSC regions' first positions were the first position for all other *Cuscuta* species (Supplementary Figure S11A-H).

*C. bonafortunae* has an inverted fragment in its SSC region (Supplementary Figure S11D). The three plastomes of *C. exaltata*, *C. japonica*, and *C. reflexa* are much longer than those of the plastomes because they contain an additional nine genes (Figure 2). *C. australis, C. obtusiflora, C. pentagona, C. gronovii and C. chinensis* show the similar structure compared with *A. thaliana* (Supplementary Figure S1 and Figure S11 I-M). In terms of the structure, *C. exaltata* has two inverted fragments with different length (Supplementary Figure S11N). In contrast, *C. japonica* and *C. reflexa* have three inverted fragments (Supplementary Figure S11O-P). The results of the structural variations are consistent with that of the phylogenetic analysis. It showed that *C. exaltata, C. japonica,* and *C. reflexa* belonged to one clade. And the rest species belonged to another clade (Figure 3).

### Molecular marker development

*C. chinensis and C. australis* are two species listed in the Chinese Pharmacopeia. They are frequently mixed up with other *Cuscuta* species due to similar morphological characters. To distinguish *C. gronovii* and two medicinal *Cuscuta* species: (*C. chinensis and C. australis*), we developed molecular markers based on their plastome sequences based on the aligned plastome sequences. In total, ten molecular markers were found (Supplementary Table S10). These molecular markers will be invaluable for the molecular discrimination of these species.

## Discussion

Here, we systematically studied the intraspecific and interspecific variations of *C. gronovii* plastomes. We analyzed the gene content, genome rearrangement, and intraspecific variations at DNA and post-transcription levels. The results painted a vivid picture of the adaptation of *Cuscuta* species to a parasitic lifestyle.

Foremost, the plastomes of all sixteen species have undergone extensive gene losses. Interestingly, these sixteen species can be divided into two groups. The first group comprises *C. australis*, *C. obtusiflora*, *C. pentagona*, *C. gronovii*, *C. chinensis*, *C. bonafortunae*, *C. camosa*, *C. boldinghii, C. mexicana, C. erosa, C. strobilacea,C. costaricensis*, and *C. chapalana*. The second group includes *C. exaltata*, *C. japonica*, and *C. reflexa*. Plastomes in the first group had ten more common genes comparing with those in the second group. Furthermore, species in the first group are closer to the outgroup taxa, suggesting that these three species in the second group had a much higher gene deletion rate. However, some genes are only missing in one species. They are likely to result from different parameters used in the annotation step. And further work is needed to confirm whether these genes are actually missing (Figure 2).

Secondly, when the eleven plastomes were compared with that of *A. thaliana*, the first group's plastomes showed 2–3 inverted regions. Examining the nine genes in group one but not in group two found that these genes are spread out in the genome (date is not shown), suggesting that the genes were deleted independently in multiple runs in the evolutionary history.

Very few data were available to explore the intraspecific variations for *Cuscuta* species. Here, we compared the two *C. gronovii* plastome sequences. In total, we found 2 INDELs and 1 SNP. The largest variation comes from an 18 insertion between the *trn*S-UGA and *psb*Z gene, accounted for 90% of the total variation identified in terms of the number of nucleotides involved. The SNP located at the *acc*D is from C to A, causing the amino acid to change from Asp to Glu. The functional consequence of this amino acid change remains to be elucidated.

RNA-editing is a post-transcriptional mechanism to modify the mRNA sequence. It will either increase the translated proteins' diversity in mitochondria or serve as an ‘error-correction’ mechanism to maintain the conserved sequences of the translated proteins in plastids. Previously, Funk et al. found 12 RNA-editing sites in *C. gronovii* (NC_009765) from the Sanger sequencing result of PCR products amplified from cDNA (Funk et al. 2007). Our scrutiny of the data suggests that only two of them (*rps*2, *rps*14) had strong signals. In this study, mapping RNA-seq data obtained from a public database to the plastome revealed two potential RNA-editing sites. Both sites locate in the rRNA coding region. This observation raises several new questions. Are these RNA-editing sites functional relevant? While future work needs to clarify the discrepancy of the RNA editing sites, it is consistent that the RNA editing level is relatively low.

The discrimination of closely related species has been vital for their correct classification and the plant materials' downstream application. Our original plan is to sequence the medicinal species *C. australis*. However, sequence comparison with those in the database suggests that our sample is most likely from *C. gronovii. C. gronovii* is a parasitic plant and widely spread in Europe and North America. Our results indicate that it might have been introduced into China by route unidentified. Regardless of what has happened, it suggests that there might be a mixed usage of different species in the production of health products involving *Cuscuta* materials. To ensure the efficacy and safety of these products, accurate identification of the source material is critical. To serve this purpose, we identified ten regions that can be used to design primers. The corresponding products can be used to discriminate *C. gronovii* and two medicinal species, *C. chinensis and C. australis,* which are listed in Chinese Pharmacopeia.

In conclusion, the identification and characterization of the plastome sequences of *C. gronovii* provide a valuable resource to analyze the intraspecific diversity among different lines of *C. gronovii* and to study the genomic evolution of *Cuscuta* species. Overall, low levels of intraspecific diversity were observed. This is possible in the context of the parasitic lifestyle of *C. gronovii*. As a parasitic plant, *C. gronovii* depends on the host cells' interaction to obtain the nutrients. As a result, having a lower mutation rate will help maintain stable interactions between the plant and its host.

## Author contributions

CL conceived the study; HMC collected samples of *C. gronovii*, MJ extracted DNA for next-generation sequencing, NY assembled and validated the genome; NY and JM performed data analysis and drafted the manuscript; PHC and LFH reviewed the manuscript critically. All authors have read and agreed on the contents of the manuscript.

## Data Availability

The genome sequence data that support the findings of this study are openly available in GenBank of NCBI at (https://www.ncbi.nlm.nih.gov/) under the accession number MT872375. The associated BioProject, SRA, and Bio-Sample numbers are PRJNA661415, SAMN16057401, and SRR12597239, respectively. The sample has been deposited in the Herbarium of the Institute of Medicinal Plant Development in Beijing, China, with the accession number: implad20170662.
